# Evolution of international collaborative research efforts to develop non-Cochrane systematic reviews

**DOI:** 10.1371/journal.pone.0211919

**Published:** 2019-02-27

**Authors:** Isabel Viguera-Guerra, Juan Ruano, Macarena Aguilar-Luque, Jesús Gay-Mimbrera, Ana Montilla, Jose Luis Fernández-Rueda, José Fernández-Chaichio, Juan Luis Sanz-Cabanillas, Pedro Jesús Gómez-Arias, Antonio Vélez García-Nieto, Francisco Gómez-Garcia, Beatriz Isla-Tejera

**Affiliations:** 1 Agencia de Evaluación de Tecnologías Sanitarias de Andalucía (AETSA), Sevilla, Spain; 2 Instituto Maimonides de Investigación Biomédica de Córdoba (IMIBIC)/Reina Sofía University Hospital/University of Córdoba, Cordoba, Spain; 3 Department of Dermatology, Reina Sofía University, Hospital, Córdoba, Spain; 4 School of Medicine, University of Cordoba, Córdoba, Spain; 5 Department of Pharmacy, Reina Sofía University Hospital, Córdoba, Spain; University of Gothenburg, SWEDEN

## Abstract

This research-on-research study describes efforts to develop non-Cochrane systematic reviews (SRs) by analyzing demographical and time-course collaborations between international institutions using protocols registered in the International Prospective Register of Systematic Reviews (PROSPERO) or published in scientific journals. We have published an *a priori* protocol to develop this study. Protocols published in scientific journals were searched using the MEDLINE and Embase databases; the query terms “Systematic review” [Title] AND “protocol” [Title] were searched from February 2011 to December 2017. Protocols registered at PROSPERO during the same period were obtained by web scraping all non-Cochrane records with a Python script. After excluding protocols that had a fulfillment or duplication rate of less than 90%, they were classified as published “only in PROSPERO”, “only in journals”, or in “journals and PROSPERO”. Results of data and metadata extraction using text mining processes were curated by two reviewers. These Datasets and R scripts are freely available to facilitate reproducibility. We obtained 20,814 protocols of non-Cochrane SRs. While “unique protocols” by reviewers’ institutions from 60 countries were the most frequent, a median of 6 (2-150) institutions from 130 different countries were involved in the preparation of “collaborative protocols”. The highest Ranked countries involved in overall protocol production were the UK, the U.S., Australia, Brazil, China, Canada, the Netherlands, Germany, and Italy. Most protocols were registered only in PROSPERO. However, the number of protocols published in scientific journals (924) or in both PROSPERO and journals (807) has increased over the last three years. *Syst Rev* and *BMJ Open* published more than half of the total protocols. While the more productive countries were involved in “unique” and “collaborative protocols”, less productive countries only participated in “collaborative protocols” that were mainly published in PROSPERO. Our results suggest that, although most countries were involved in solitary production of protocols for non-Cochrane SRs during the study period, it would be useful to develop new strategies to promote international collaborations, especially with less productive countries.

## Introduction

Systematic reviews (SRs) and meta-analyses (MAs), the standards for evidence synthesis of primary studies, are extremely useful for supporting decision making processes in the context of Health Systems [1]. However, it is necessary to ensure that these decisions are supported by reviews of the highest methodological quality and that they show the lowest risk of bias [2]. The Cochrane Handbook for Systematic Reviews of Interventions states that a protocol should be prepared before publishing an SR [3]. The 2010 Preferred Reporting Items for Systematic Reviews and Meta-Analyses (PRISMA) statement called for the registration of SR protocols [1, 2]. Preparing an *a priori* protocol reduces the potential for bias in the review process and increases the transparency of the analysis and the results [4]. Furthermore, making the content of a protocol available for public access could also reduce duplication [5, 6], chances of peer review before starting the review process, and audit discrepancies between the protocol and the finally produced SR [7–9].

Bias limits the possibility of finding or identifying the actual outcomes of a given research activity. Rigour adherence to the scientific community’s established methodological agreements minimizes bias and reduces uncertainty about the estimates we make. One of the proposed methods to reduce bias in SRs is to develop a comprehensive protocol that contains 1) the sources of primary data; 2) procedures for searching, extracting, filtering, selecting, and analyzing data; and 3) analytical and methodological tools that will be used to conduct the research. For now, it is only recommended that such protocols should be prepared before making the first analysis and that they can be consulted at any time, leaving the trace that were elaborated much before the final results were published [10].

Therefore, a protocol should have two main features: first, it should contain all necessary instructions to reproduce the same results from the respective SR; second, it should be prepared before the SR is conducted. Currently, a protocol can be freely viewed by the scientific community, and this allows for the functionality of tracking dates to ensure that the protocol has been created prior to the review: public repositories and scientific journals provide these functionalities. PROSPERO (International prospective register of systematic reviews) is an international database for prospectively registered systematic reviews; it is funded by the National Institute for Health Research (NIHR). Scientists worldwide use the database, and within less than a decade, it has reached a landmark achievement of 30,000 registrations. The database is free and open for all researchers who are planning to conduct an SR and also for those who are searching for registered, ongoing, or completed reviews in order to develop meta-epidemiology studies. The PROSPERO Advisory Group Statement of Founding Principles centers on free access to both registration and searches on the database; it is as inclusive as possible and achieves the key aims of avoiding duplication and minimizing bias in SRs.

The second option is to publish the protocol in a scientific journal. This option allows peer reviewers and editors to assess the quality of the protocol based on scientific criteria. Through their comments and suggestions, reviewers may suggest changes to improve methodological quality. However, there is still no empirical evidence from meta-epidemiological studies that these changes in protocol will be crucial for improving the quality of SRs. There are many journals that accept protocols of SR for publication. Some of them are *BMJ Open* (https://bmjopen.bmj.com/) and *Systematic Reviews* (https://goo.gl/mFShxv).

No matter where protocols are published, a descriptive analysis of these documents can give us a glimpse of the efforts made by researchers to carry out SRs. Allers *et al*. have recently found that one third of these protocols remained unpublished after 3-5 years, and this could be an unknown source of bias not studied so far, similar to those meant for many randomized clinical trials (RCTs) that were registered in public repositories such as ClinicalTrials.gov but never been published [11]. In a first approximation to this problem, we aimed to describe comprehensively how different reviewers represented by the institutions and countries to which they belong, make efforts to design these protocols and their strategies to conduct reviews.

To date, no study has formally assessed the relationships between reviewers, represented by their respective institution or country, when elaborating an *a priori* protocol to develop a non-Cochrane systematic review, analysing co-working patterns, and evolution of strategies to make these protocols publicly accessible.

## Materials and methods

### *A priori* published protocol

We published an *a priori* protocol in *Systematic Reviews* [12]. This protocol describes source of data, methods to perform document searches (PROSPERO web scraping and literature databases queries), eligibility and screening, data extraction, analysis, and the reporting of results.

### Web scraping and literature search strategies

Records stored in PROSPERO (https://www.crd.york.ac.uk/prospero/) were obtained by using a custom Python 3.0 script for web scraping, and Chrome’s Web Scraper website data extraction tool (http://webscraper.io/) was used for automatic and iterative extraction of raw data from all the completed non-Cochrane registration records stored from February 2011 to December 2017. More detailed information about scraping methods is available in Supporting Information file (S1 File). Protocols published in scientific journals were obtained by querying MEDLINE and Embase using the *RISmed* R package and the Boolean terms combination ‘[“Systematic Reviews” [Title] AND “protocol” [Title]]’.

### Data filtering and eligibility criteria

Registers or protocols with a less than 90% section fulfilment rate and those that were duplicated (i.e., those sharing titles and reviewers) were dropped from the dataset. An R script automatically performed the screening process. Subsequently, the results were subjected to human verification by two reviewers (JG-M and MA-L) to check for inconsistencies between machine and human tasks. In that case, human decision was adopted.

### Dataset and variables

A working .csv file, which included only the variables that we wished to subject to further analysis, was obtained (Supplementary methods). Protocols with different reviewer’s affiliation countries were considered to be the result of international collaboration, and their respective countries were analyzed because they co-appeared in the protocol as “unlisted” and tagged as contributing to “Collaborative protocols”. Protocols with unique reviewers’ affiliation country were considered to be produced by a unique country and were tagged as “Unique protocols”. More detailed information about dataset and variables is available in Supporting Information file (S1 File).

### Demographics and the evolution of protocol production

Considering the source (journal vs. PROSPERO), type of journal, and country, some panels from the plots were considered to represent changes in the number of protocols published from 2011 (the year PROSPERO was launched) to 2017 (the year PROSPERO web scraping was performed. Considering the entire list of affiliation-association countries for all co-reviewers per protocol, we displayed a world map that used different colors to represent the number of times any country was involved in any protocol.

### Data visualization and statistical analysis

Qualitative variables were summarized by frequency level (number, percentage) or displayed using several types of graphs (mosaic plots, density/histogram plots, etc.). Quantitative variables were summarized using the mean (standard deviation) or median (interquartile range) for non-normally distributed variables. Graphs were produced, and statistics were analyzed using several R 3.4.4 language packages [R Development Core Team(http://www.R-project.org]; the Venn diagram was obtained using the eulerr shinny app (http://eulerr.co), and the workflow figure was created using Review Manager 5 (RevMan 5) software (https://community.cochrane.org/help/tools-and-software/revman-5). Our analysis can be fully reproduced by using several source files containing raw data, and R scripts are available in the form of an R notebook (https://github.com/info4cure/PROSPERO_protocols_demographics). It is shared as an open source software under the MIT license. A Python script for PROSPERO web scraping will be publicly released by the end of 2018, once our team completes all the analyses related to the main project.

### Protocol vs. research-on-research study

Our planned search strategy was published in *Systematic Reviews* journal and compared with the final reported review methods from this study. The methods for web scraping, filtering, and selection did not change. However, this is the first article that we have produced for this research project, and the project constitutes only a partial descriptive analysis compared to the main goals described in the above-mentioned protocol.

### Ethical considerations

Since our study did not collect primary data, formal ethical assessment and informed consent were not required.

## Results

### Search results

**Fig 1:**
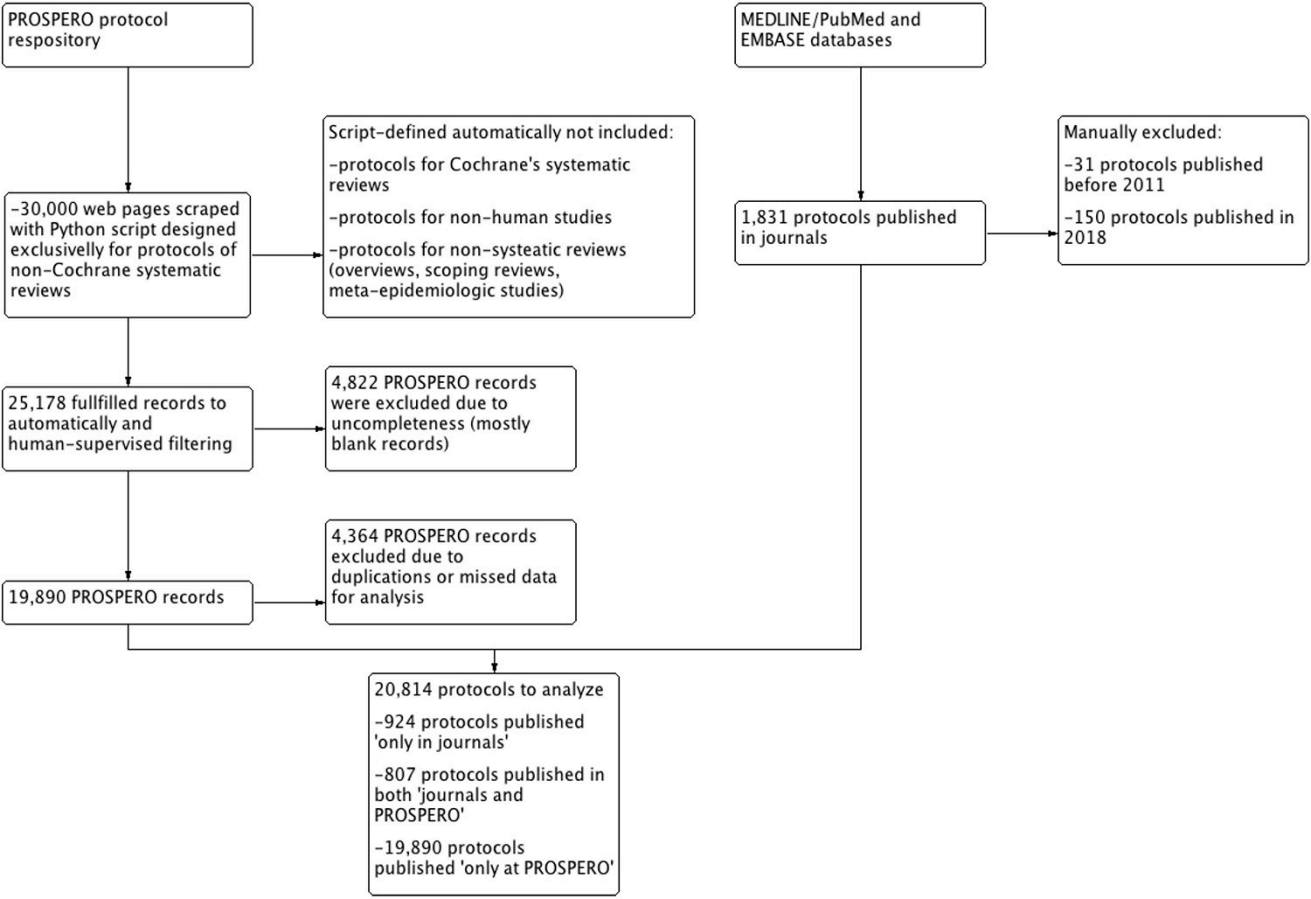
PRISMA workflow for searching for PROSPERO records and protocols published in scientific journals with regard to non-Cochrane systematic reviews.

**Fig 2:**
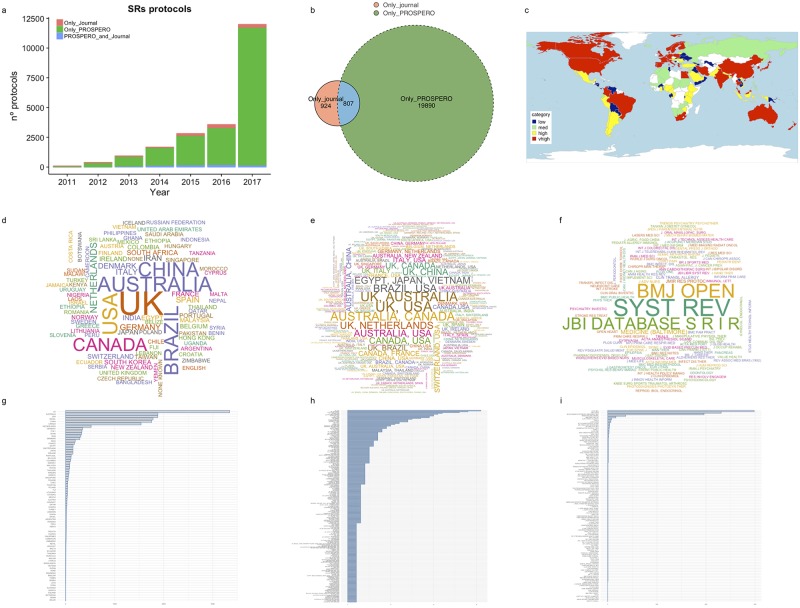
This panel represents the main features of included protocols. (a) The frequency of protocols published from 2011 to 2017 compared to those published “only in journal”, “only in PROSPERO”, and “journal and PROSPERO”. (b) Venn diagram of the number of protocols published “only in journal” (coral), “only in PROSPERO” (green), and “journal and PROSPERO” (blue)—their intersection. (c) Map representation of the number of protocols produced categorized by country (as a proxy for the reviewer’s affiliation country). Colors represent the levels of productivity, as defined by the quartiles of a newly recoded variable [abs(log2(country.count / all.countries.count))] (red, very high; yellow, high; green, medium; blue, low). (d), (e), and (f) represent the word clouds of “unique countries” (d), “collaborative countries” (e), and journals (f). Text size and centering is proportional to the associated number of protocols. Colors have been randomly assigned. (g), (h), and (i) represent column plots of the “unique countries” (g), “collaborative countries” (h), and journals (i), which are ranked based on the total number of protocols.

After scraping 30,000 PROSPERO records, 5,362 documents were excluded because they were 118 not fulfilled, and 903 were duplicated versions of other protocols (Fig 1). After text mining and manually supervising the obtained dataset, 4,364 protocols were excluded because they were lacking crucial information required for the analyses. By searching bibliographic databases, we obtained 1,732 protocols of SRs published in scientific journals. Only 807 protocols were shared by both PROSPERO and the journal datasets (Fig 2b).

### General characteristics

Thus, 20,814 protocols published from 2011 to 2017 were finally included for further analyses. These protocols comprised 10,888 reviewers affiliated to institutions across 130 countries. The median number of reviewers and institutions per protocol was five in the ranges 1–57 and 1–42, respectively. The total number of produced protocols increased—following an exponential pattern—from 2011 to 2017, mainly due to those registered “only in PROSPERO” (Fig 2a); these were distantly followed by protocols published “only in journals” or published in “journal and PROSPERO” (Fig 2b).

### Scientific journals vs PROSPERO repository

**Fig 3:**
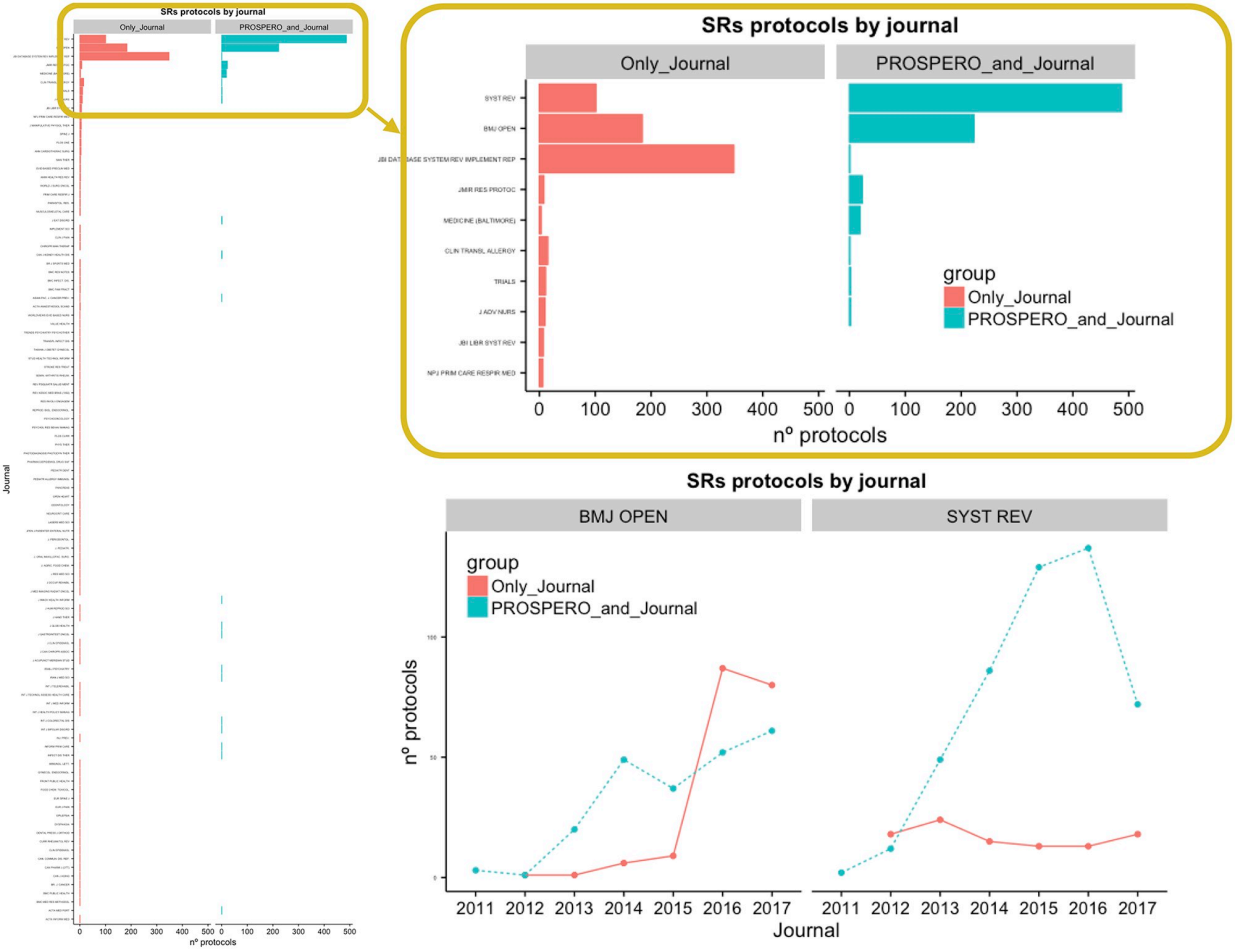
This panel represents the frequency and time-course changes of SR protocol publication by journals. (a) Frequency of protocols published from 2011 to 2017 by journals compared the protocols published “only in journal” with protocols published in “journal and PROSPERO”. (b) Magnified version of the plot (a) centered on the top 10 most published journals. (c) Evolution of “only in journal” vs. in “journal and PROSPERO” protocol publications from 2011 to 2017 compared to “BMJ Open” and “Systematic Reviews” journals. SR: *Systematic Review*. *SR: Systematic Review*.

**Fig 4:**
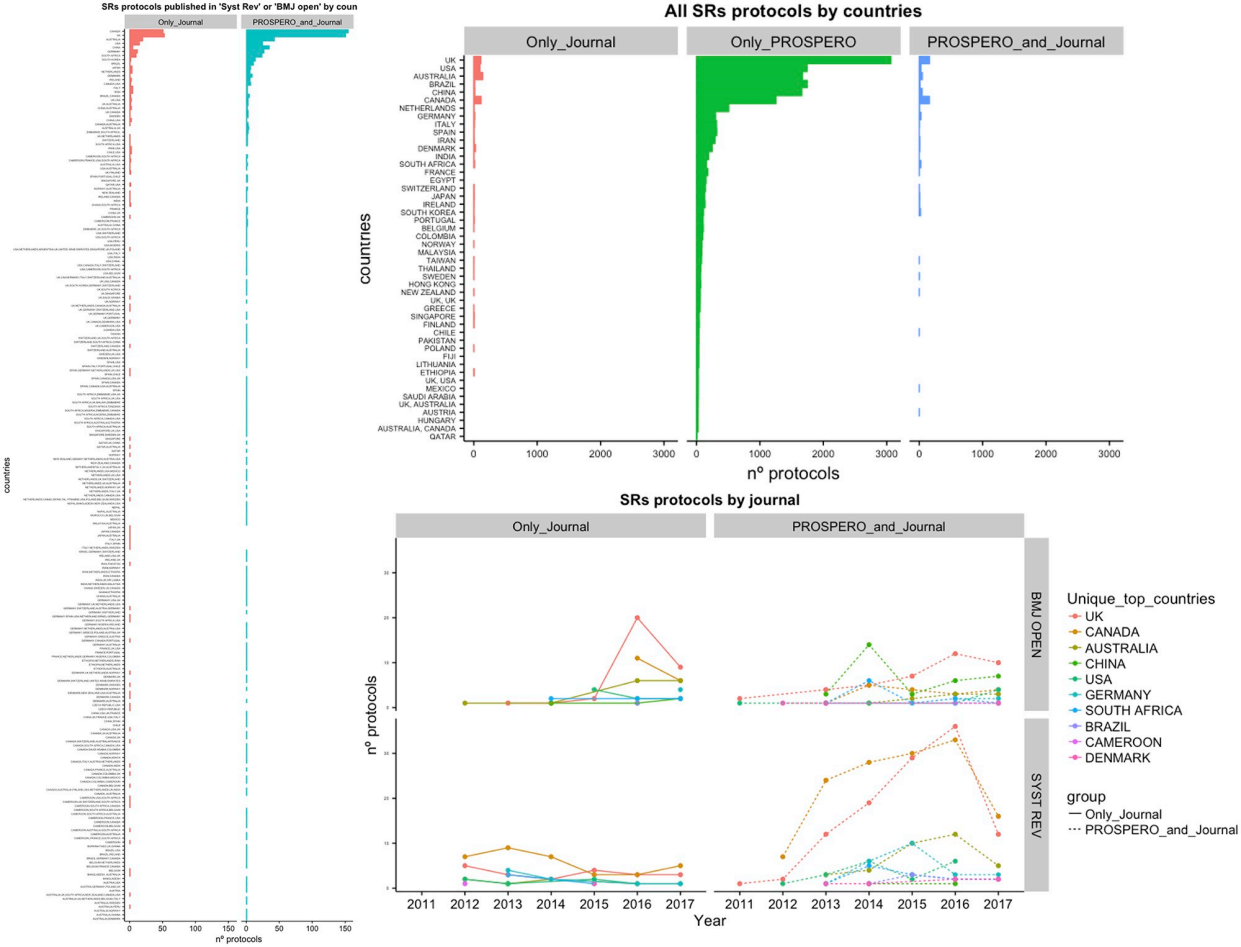
This panel represents the frequency and time-course changes of SR protocol publications categorized by countries. (a) The frequency of protocols published from 2011 to 2017 categorized by country comparing protocols published “only in a journal” with protocols published in “journal and PROSPERO”. (b) Magnified version of the plot (a) centered on the top 10 most productive countries comparing protocols published “only in a journal”, “only at PROSPERO”, and in both “journal and PROSPERO”. (c) Evolution of “only journal” vs. “journal and PROSPERO” protocols publications from 2011 to 2017 comparing the top 10 most productive countries.

There were 124 journals where 1,758 protocols of SRs were published (Fig 3a). Some of these journals (ranked by frequency order) were “BMC Systematic Reviews” (Syst Rev), “British Medical Journal Open” (BMJ Open), “JBI Database of Systematic Reviews and Implementation Reports”, “Medicine (Baltimore)”, “JMIR Research Protocols”, “Clinical and Translational Allergy”, and “TRIALS” (Figs 2f, 2i and 3a). More than half of all the protocols published in scientific journals are available in “Syst Rev” (33%) or “BMJ Open” (27%) (Fig 3b). The “Syst Rev” published the maximum number of protocols during 2011-2017, with the majority (80%) also being registered at PROSPERO (Fig 3b). This publishing strategy has increased considerably since 2011, while the number of protocols without PROSPERO registration remained consistently low during the study period (Fig 3c). This trend seems to be associated with the major numbers of protocols authored by reviewers affiliated with institutions from the UK and Canada and, to a lesser extent, Australia (Fig 4c). On the contrary, “BMJ Open”, the second journal with a higher number of published protocols seems to follow a different pattern of protocol publication. First, the same number of published protocols was identified for with vs. without PROSPERO registration (45%/55%) for the entire study period (Fig 3b). Second, there was a shift in publication patterns during 2015: published protocols that were also registered in PROSPERO formed the majority for the first period (2011-2015), with a peak in protocols from China in 2015 (Fig 4c). However, since 2016, published protocols without PROSPERO registration have become more frequent, and such protocols have been mainly produced by institutions in the UK (Fig 3c).

### Protocols by reviewers’ affiliation countries

In the case of most of the countries, reviewers belonging to institutions had participated in at least one protocol (Fig 1c). The distribution levels of each country’s participation are depicted in Fig 3.

**Fig 5:**
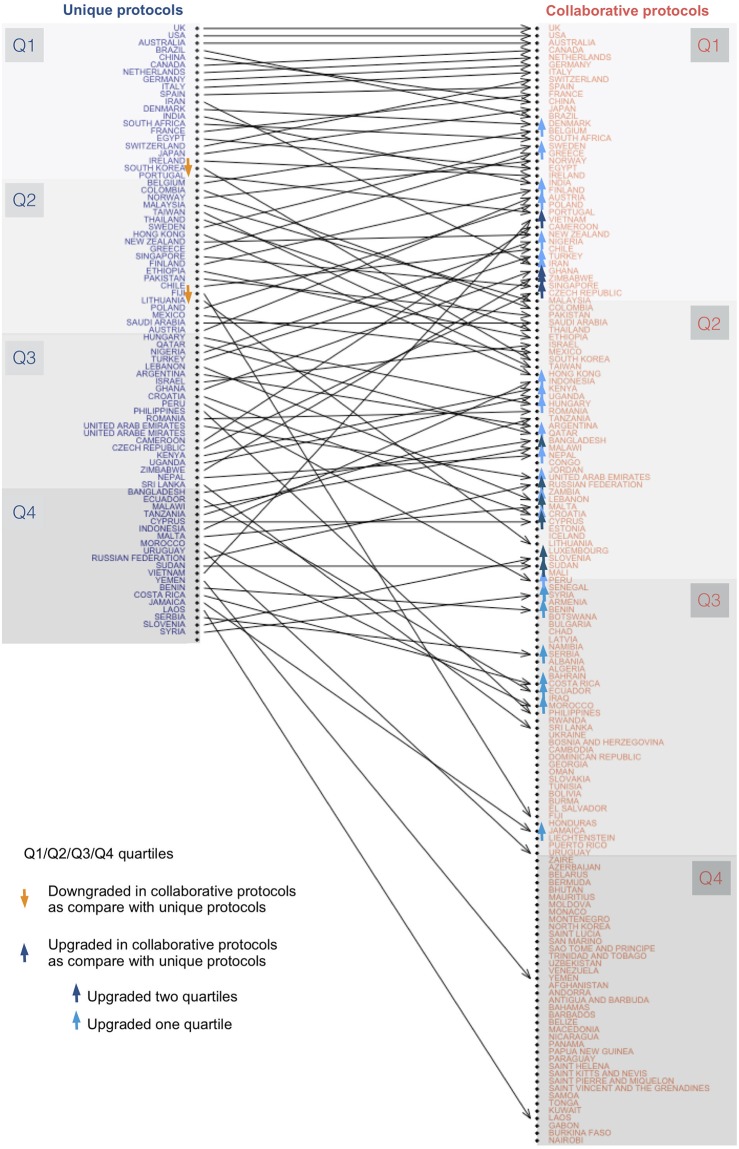
Rank discrepancies between two ordered lists of reviewers’ affiliation countries. The “Unique protocols” column displays a descending list of reviewers’ affiliation countries, which produced protocols in which all reviewers’ institutions belonged to a unique country. The “Collaborative protocols” column displays a ranked list of reviewers’ affiliation countries that collaborated with other reviewers’ affiliation countries to produce protocols for SRs. Arrows connect the same country from the first list to the second list. Countries represented only in one of the lists are not connected to/by any arrow. The countries were sub-grouped (Q1:Q4) by cutting through 25%, 50%, and 75% of the total number of countries in each list. When comparing the “Unique protocols” and “Collaborative protocols” lists, the country position was considered as being modified if the edge connected two different subgroups (i.e., Q1→Q3). *Direction of the change defines ′upgrading′* (*Q*2 → *Q*1, *Q*3 → *Q*1, *Q*4 → *Q*1, *Q*3 → *Q*2, *Q*4 → *Q*3) *or ′downgrading′ the rank position of any country* (*Q*1 → *Q*2, *Q*1 → *Q*3, *Q*1 → *Q*4, *Q*2 → *Q*3, *Q*2 → *Q*4, *Q*3 → *Q*4).

Despite regional differences (because of the involvement of institutions from around the world), the generation process of protocols has been very similar. However, we show that African countries-in comparison with other countries-produced the lowest numbers of protocols. From a total of 90 countries, we found that most of the protocols (17,431; 90%) were authored by reviewers from institutions from a single country (31.5% of countries worldwide). In contrast, a few protocols (1,938) were elaborated by researchers who collaborated with institutions from two or more countries (from a list of 130) (45.62% of the 285 countries selected) (Fig 5). The word clouds (Fig 1d–1f) and bar diagrams (Fig 1g–1i) show the predominant participation of institutions from countries such as the UK, the U.S., Australia, Brazil, Canada, China, and the Netherlands with regard to producing protocols either in isolation or in collaboration with others countries (Figs 1e, 1h and 4b).

The production of collaborative protocols has increased in recent years. These protocols have been published in PROSPERO since 2011, and later, these protocols were also published in journals (2013); some collaborative protocols were published only in journals (2014). Protocols from a greater number of countries (more than 30) have appeared since 2016, with more than 150 different countries participating in producing protocols in 2017. The list of countries ranked by participation in collaborative protocols is characterized by the following attributes: a) larger than the list of countries producing unique protocols; b) the top 10 countries are repeated in both lists (except China and Brazil, which were replaced by the Netherlands and France in the collaborative list); c) almost all the countries involved in producing “unique protocols” participated in the creation of “collaborative protocols”, as this is more productive than working in isolation; d) countries that only participated in collaborative protocols were the least productive (Fig 5).

### Analysis of time-course patterns

**Fig 6:**
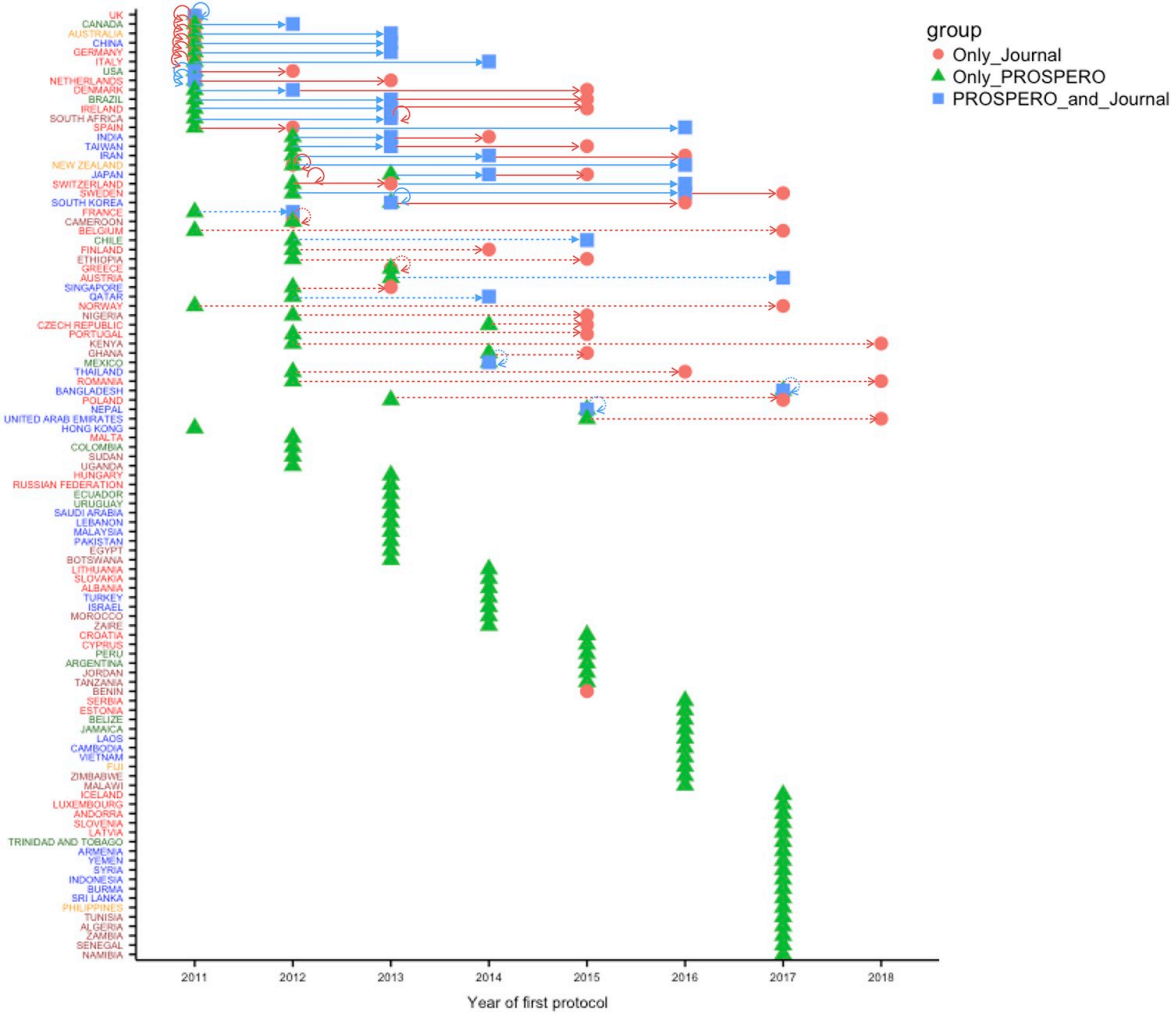
Analysis of protocol publication patterns categorized based on the most productive countries. Countries are listed in a descendent order based on their “Unique protocol” productivity. The points represent a hallmark in every country’s history of protocol publication: first publication of a protocol in “only in journal” (red dot), “only in PROSPERO” (green triangle), and in “journal and PROSPERO” (blue square). Arrows connect two (by a dotted line) or more (by a full line) hallmarks to emphasize how much time a country requires to adopt a new publication process.

Fig 6 displays “the year of the first protocol published” by “source of publication” (“only in PROSPERO”, “PROSPERO and journal”, “only in journal”) and by “country”. To simplify the analysis, only protocols without collaborations between countries were considered for this plot. There seem to be four different patterns. The first pattern shows that the most productive and collaborative countries (the UK, Canada, Australia, China, Germany, Italy, the U.S., and the Netherlands) started producing protocols very early and submitted them to PROSPERO or published them in journals; however, it was only after 1-3 years that they started publishing protocols in both PROSPERO and journals. The second pattern is as follows: some countries (Denmark, Brazil, Ireland, South Africa, Spain, India, Taiwan, Iran, New Zealand, Japan, Switzerland, Sweden, South Korea, and France) started publishing their protocols in PROSPERO and, after 1-3 years in PROSPERO and journals, and finally, after another 1-4 years, they finally started publishing their protocols only in journals. The third pattern defines countries (from Belgium to United Arab Emirates) that started using only PROSPERO, not too early, and most of them, after a longer period of 3-5 years, published only in journals without submitting their protocols to PROSPERO anytime. In the final pattern, least productive countries covering from Hong Kong to Namibia, and representing more than half of the world’s countries, submitted their protocols only to PROSPERO.

## Discussion

### Main findings

This is the first research-on-research study that describes the diversity of collaborative strategies and time-course preference changes adopted by countries whose institutions are involved in producing and communicating protocols for SRs. John P. A. Ioannidis defines research-on-research or meta-research studies as the study of research itself: its methods, reporting, reproducibility, evaluation, and incentives [13]. Overall, our findings suggest three observations: first, most countries are involved in producing protocols for SRs, although the majority of protocols are produced without international collaboration; second, although most protocols were earlier registered only in PROSPERO, this tendency seems to have changed since 2013-2014, and most productive countries have now begun to publish protocols mainly in “Syst Rev” or “BMJ Open” journals; third, less productive countries participate in collaborative international protocol production, and such protocols are predominantly submitted to PROSPERO and not to scientific journals.

### Our findings in context

Our results show that most protocols for non-Cochrane SRs were authored by reviewers from institutions belonging to a single country. As most of the topics are not restricted by local, ethnic, or geographic factors, this approach may become a challenge for transforming evidence into practice worldwide. Collaborations between countries, especially more vs. less productive institutions, may enhance technical expertise by providing training to the latter regions and extending collaboration beyond SRs, thus improving the adoption of evidence-based health policies, selecting the best evidence for the right audience, and increasing focus on relevant issues through appropriate methodologies [14]. This will be possible with the growing innovation in tools and platforms that could enable more efficient SR production through collaboration.

The registration forms of protocols submitted to PROSPERO are only checked against the scope for inclusion in the repository and for clarity of content. Once accepted, an audit trail detailing the major changes to planned methods may be checked at any time, even after the SR is published. Ideally, registration should take place before the researchers start formal screening against inclusion criteria, but reviews are eligible as long as they have not progressed beyond completing data extraction. However, accomplishing these goals is still determined by the reviewers’ integrity. Dawid Pieper and Katharina Allers recently suggested that an *a priori* design of an SR may not have the same advantages and potential to reduce the risk of bias in SRs compared to those of RCTs. They argue that “living SRs”, involving new workflows and collaboration tools, text mining and machine learning technologies, emerging reusable data repositories, shared ontologies, and harmonized data transfer protocols can be expected to decrease any potential manipulations in the future.

Since 2011, there have been few meta-epidemiological studies about the content and use of PROSPERO. A descriptive analysis of the number of PROSPERO registrations and the website usage from 2011 to 2017 has recently been published, and it explores the epidemiological characteristics and completeness of pre-specification primary outcomes in a small sample of PROSPERO records [15]. They highlight the exponential increase in registered protocols at PROSPERO between 2011 and 2017. However, these authors recognize that there are still many caveats regarding the real utility of making a document, which has not been reviewed methodologically, available for public access. These authors—one is a member of PROSPERO’s international advisory group—raise three issues that will certainly generate future debate about new strategies for future improvements for PROSPERO: how closely do published SRs adhere to the planned methods of PROSPERO registrations?; can the specification of greater outcomes in PROSPERO registrations prevent inclusion and reporting biases?; and do registered SRs address the necessary question with regards to developing the review?

### Limitations and strengths

Our study analyzed the largest sample of SR protocols produced in the last seven years. We did not perform PROSPERO registration record sampling [15]. Rather, our objective was to obtain the entire universe of registers (from the first document to the last one registered just before the date selected for web scraping), not representative samples of them. The search specificity for non-Cochrane PROSPERO registration records was based on a Python script that was designed to recognize only the format of these records; this differs from registration records for Cochrane and non-human studies. These cannot be scraped using our script due to the structural differences in PROSPERO forms. Thus, the sensitivity and specificity for the web scraping is 100%.

The present study, however, also had several limitations. First, we used countries as proxies for reviewers or reviewers’ institutions. Better size of information granularity would have enabled deeper analysis regarding the reviewers’ and institutions’ productivity and collaborative networks. However, based on technical issues related to the variety of formats used by reviewers when fulfiling PROSPERO forms and time limitations on the project, we decided to use countries as the unit of analysis. We have used this approach previously to analyze how the author-paper affiliation network architecture influences the methodological quality of SRs and MAs for psoriasis [16]. Second, our study is limited to protocols in non-Cochrane SRs. Cochrane Reviews are demonstrated to have better methodological quality and lower bias risk than non-Cochrane SRs [17, 18]. The Cochrane Database of Systematic Reviews (CDSR) is the leading resource for Cochrane SRs and protocols. Protocols for Cochrane Reviews have also been published in PROSPERO since 1 October 2013. This fact introduces a publishing gap at PROSPERO, with only non-Cochrane SRs being published from 2011 to 2013. Another area of particular concern in relation to non-Cochrane reviews is the failure to register reviews at the outset. Registration of Cochrane reviews is mandatory with publication of a protocol *a priori*. To avoid an unbalanced sample of protocols with different proportions of quality and rates of publication, we decided to select only non-Cochrane SRs protocols for our study.

### Implications of results

Future work should focus on analyzing co-authorship networks. This would help to identify academic talent, integrate the research interest of experienced researchers and local investigators who can recognize regional resources and health-care necessities that change over time, thus increasing opportunities for improving international collaborations. A recent article that mapped 115,000 RCTs has demonstrated the mismatch between research efforts and health needs in non-high-income regions [19]. Similarly, implementing strategies is necessary for the efficieny coordinatioe of collaborations between countries to perform non-Cochrane SRs, and this is especially so when most human resources and stakeholders are not coordinated by a consolidated international organization such as the Cochrane.

Our results demonstrate that most protocols are registered only in PROSPERO. However, interested reviewers should consider these facts: PROSPERO registrations cannot be methodologically curated, critically peer-reviewed, or freely commented upon by anonymous readers. Whether protocols should be submitted *a priori* to a public repository and/or to a journal is debatable. Indeed, our data show that this tendency has been changing since 2013-2014, and most productive countries have begun publishing protocols in “Syst Rev” or “BMJ Open”.

Future studies comparing the methodological quality of protocols registered “only in PROSPERO”, “only in journals”, and in “PROSPERO and journals” should provide empirical evidence for an *a priori* peer-reviewing process of protocols before authors start SR development. Furthermore, it would be interesting to explore whether the modifications suggested by peer-reviewers after submitting an SR protocol to a journal significantly improves the quality of protocols (i.e., assessment by using PRISMA for Protocols extension), and even to increase the methodological quality and reduce the RoB in the final SR would be of great interest.

## Conclusions and future research

Although most countries worldwide were involved in producing protocols for non-Cochrane SRs, it is important to develop new strategies to boost international collaborations, especially between more productive and less productive countries. While most protocols of SRs are submitted to PROSPERO, the potential advantage of the new trend of publishing protocols in scientific journals and not only in PROSPERO should be evaluated further.

## Supporting information

S1 File (DOCX)
